# Evaluating Features and Variations in Deepfake Videos Using the CoAtNet Model

**DOI:** 10.3390/jimaging11060194

**Published:** 2025-06-12

**Authors:** Eman Alattas, John Clark, Arwa Al-Aama, Salma Kammoun Jarraya

**Affiliations:** 1 Computer Science Department, Faculty of Computing and Information Technology, King Abdulaziz University, Jeddah 21589, Saudi Arabia; smohamad1@kau.edu.sa; 2 School of Computer Science, University of Sheffield, Regent’s Court, Sheffield S1 4DP, UK; john.clark@sheffield.ac.uk; 3 Institutional Advancements, King Abdullah University of Science and Technology, Thuwal 23955, Saudi Arabia; arwa.alaama@kaust.edu.sa

**Keywords:** digital multimedia forensics, deepfake, Generative Adversarial Networks (GANs), computer vision (CV), CoAtNet

## Abstract

Deepfake video detection has emerged as a critical challenge in the realm of artificial intelligence, given its implications for misinformation and digital security. This study evaluates the generalisation capabilities of the CoAtNet model—a hybrid convolution–transformer architecture—for deepfake detection across diverse datasets. Although CoAtNet has shown exceptional performance in several computer vision tasks, its potential for generalisation in cross-dataset scenarios remains underexplored. Thus, in this study, we explore CoAtNet’s generalisation ability by conducting an extensive series of experiments with a focus on discovering features and variations in deepfake videos. These experiments involve training the model using various input and processing configurations, followed by evaluating its performance on widely recognised public datasets. To the best of our knowledge, our proposed approach outperforms state-of-the-art models in terms of intra-dataset performance, with an AUC between 81.4% and 99.9%. Our model also achieves outstanding results in cross-dataset evaluations, with an AUC equal to 78%. This study demonstrates that CoAtNet achieves the best AUC for both intra-dataset and cross-dataset deepfake video detection, particularly on Celeb-DF, while also showing strong performance on DFDC.

## 1. Introduction

Videos are an extremely common multimedia form that can be conveniently transferred to different social media platforms, such as WhatsApp, YouTube, Instagram, and Facebook. Multimedia content, including videos, can be readily modified via modern editing tools [1]. This is perhaps the start of a slippery slope regarding the “authenticity” of such content. Modifications may have positive aesthetic or presentational goals, but, of course, some modifications may be intended to mislead. Where videos are concerned, such matters took a significant turn in 2017 when a Reddit account called “Deepfake” posted synthetic pornographic videos generated using a Deep Neural Network (DNN). The account’s name, combining “Deep” (from deep learning) and “fake”, caught on and now refers to hyper-realistic images, speech, and videos generated using Generative Adversarial Networks (GANs) that render the identification of their authenticity difficult for humans [2,3].

While digitally synthesising faces or manipulating a real face requires a significant volume of source data, such data are now publicly available. Contemporary deep learning techniques, such as autoencoders (AEs) and Generative Adversarial Networks (GANs), eliminate several manual editing processes [4].

Multiple mobile applications, websites, and software programs have been made publicly available, allowing for the production of high-level synthesised media. These resources require no prior training. Applications such as FakeApp [5], DFaker [6], Faceswap-GAN [7], Faceswap [8], and DeepFaceLab [9] have been used to create the deepfakes contained in deepfake datasets or videos circulated on the Internet that involve celebrities, such as former president Barack Obama [10] and actor Tom Cruise [11].

Although there is concern regarding deepfake technology, it also has creative and productive applications [4,12,13,14]. For example, it can be used in education, criminal forensics, virtually trying on clothes while shopping, 3D modelling industrial applications, entertainment [15], acting [16], film production, and video dubbing [17]. In education, deepfakes can enhance engagement by transforming teachers into familiar characters or animating historical figures for classroom interaction. In privacy and healthcare, they can help to de-identify patients in videos while preserving useful visual information and serve as virtual counsellors, and are especially effective for individuals with social anxiety. Additionally, AI characters can play roles in preserving culture and history by bringing historical artworks and figures to life. These technologies can enrich storytelling, therapy, and cultural preservation efforts [18].

At the same time, deepfakes have raised significant concerns, particularly due to the potential for their abuse and misuse [2,19,20]. Deepfake videos can misrepresent a person’s views and actions, which could result in serious political, social, financial, and legal issues. Deepfakes pose wide-ranging threats if used harmfully: the manipulation of the stock market, political discourse, or elections; targeting celebrities with revenge porn; creating fake news and spreading misinformation; financial fraud; and creating fake social media accounts to incite violence or direct the public to specific perspectives. These fake multimedia have serious consequences, such as misinforming the public, harming a personal or business reputation, affecting political perspectives, and being maliciously used as evidence in court.

Various laws seek to protect individuals against the misuse of deepfake technology. For example, in the USA, the DEEPFAKES Accountability Act (H.R. 5586) [21] establishes civil remedies for victims of harmful deepfake content, giving victims the right to initiate civil actions against individuals who create or distribute deepfake material that causes harm. This empowers victims to seek damages and injunctive relief, offering a legal avenue to address and mitigate the impacts of malicious deepfake content. There are also similar regulations in Canada [22] and China [23].

Deepfake misuse leads to doubts about available videos and concerns about people’s privacy [24]. Moreover, it poses an issue for security and ethics, as visual media can no longer be considered trustworthy content [25]. Consequently, there is a great demand for methods to verify that videos are genuinely what they appear to be.

As the public’s interest in deepfake technology grows, so will the number of relevant studies. Over the last three years, tremendous progress has been made in developing detection technologies. The academic community, research groups, and commercial companies worldwide are undertaking relevant studies to mitigate the negative effects of such a problem [26].

Most research to date has involved training and evaluating detection models using a restricted dataset. The training dataset will contain application instances of the same faking techniques, perhaps in addition to the same real-world environments. Models developed in this way often exhibit good generalisation performance on “unseen” examples, but they can radically underperform when such data instance assumptions are relaxed. For example, a model trained using a dataset that includes fakes made using three different techniques may be useless at detecting fakes that are created using a fourth technique. In a sense, models are developed to carry out generalisations in an intra-dataset manner, but they do not generalise reliably across datasets (i.e., when applied to different datasets).

In this study, we investigate the generalisation capability of the CoAtNet model for deepfake video detection across multiple datasets. The CoAtNet model combines aspects of convolutional networks and specific vision-focused attention networks known as Vision Transformers. While CoAtNet has demonstrated exceptional performance in various computer vision tasks, its effectiveness in distinguishing real from manipulated videos—particularly in cross-dataset scenarios—remains underexplored. Our research systematically evaluates CoAtNet’s performance using benchmark deepfake datasets, including FaceForensics++ [27], DFDC [28], Celeb-DF [29], and FaceShifter [30], to assess its robustness and adaptability to unseen data. The main contributions of this study can be summarised as follows:

- We evaluate the generalisation ability of the CoAtNet model in deepfake videos for synthesised faces and discover different features and variations.
- Our study proposes an improved CoAtNet model (CoAtNet16A) that ensures better generalisation.
- We investigate the detection effect of CoAtNet16A using different frame selection strategies, including a single middle frame, fifteen random frames, fifteen optical flow frames, cosine similarity keyframes, and facial landmark keyframes.

Our method—using CoAtNet with a voting-based approach that integrates predictions from single frames, random frames, and optical flow frames—achieved outstanding performances on the FF++ dataset, with an AUC of 0.9996, surpassing leading methods. In cross-dataset evaluations, our model demonstrated superior results on the Celeb-DF dataset with an AUC of 0.76 and on the DFDC dataset with an AUC of 0.68 (the fake images in these datasets were created using different manipulation techniques).

The remainder of this study is organised as follows: Section 2 reviews related research on deepfake generation and detection, including CNN-based approaches, Vision Transformer (ViT)-based approaches, and the CoAtNet model. Section 3 illustrates the two-stage proposed methodology and the details of the experiments. Section 4 outlines the comparison of performance evaluations for both intra-dataset and cross-dataset contexts. Finally, Section 5 provides the conclusions, limitations, and future research directions.

## 2. Related Research

In recent years, extensive research has been conducted to address the growing challenge of deepfake detection, driven by the rapid advancements in deepfake generation techniques. Early detection methods primarily relied on machine learning techniques and then on Convolutional Neural Networks (CNNs) due to their strong ability to capture spatial features from images and videos. However, with the emergence of more sophisticated and realistic deepfakes, researchers have explored advanced architectures such as Vision Transformers (ViTs), which excel at modelling global dependencies within visual data. Recently, CNN/ViT hybrid models such as CoAtNet have been introduced. This section summarises deepfake generation techniques, highlighting the strengths and weaknesses of both CNNs and ViTs, and explains how the CoAtNet model combines them.

### 2.1. Deepfake Generation

There are several techniques to generate hyper-realistic images, videos, and audio. However, the most used techniques are variations or combinations of deep learning architectures, such as Encoder–Decoder networks and Generative Adversarial Networks (GANs) [31]. Encoder–Decoder (ED) networks consist of an encoder that extracts latent features from an image and a decoder that reconstructs the image from these features [12]. On the other hand, a GAN comprises two competing neural networks: a generator G and a discriminator D. G produces fake samples to deceive D, while D learns to distinguish between real samples and fake samples. The repetition of this scenario results in G developing better samples (i.e., they increasingly cannot be distinguished from those of the real samples) [32].

**Figure 1:**
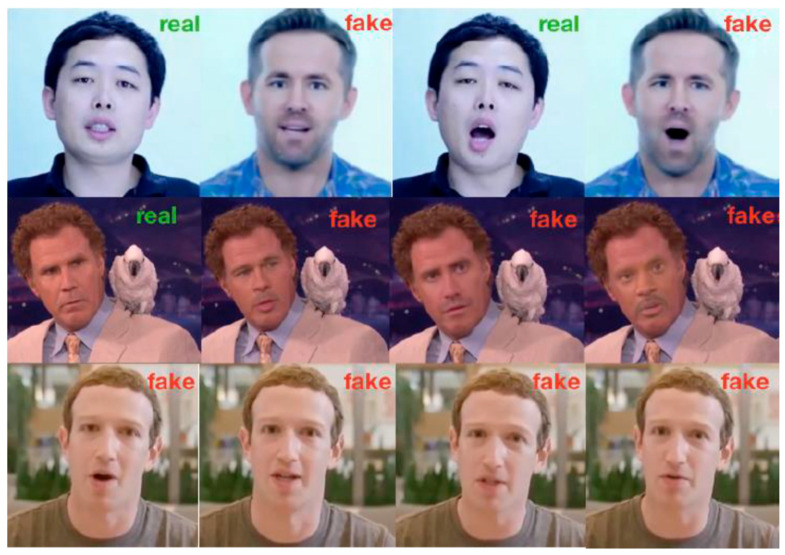
Deepfake manipulation types: (**top**) head puppetry, (**middle**) face swapping, and (**bottom**) lip syncing. Source: [2].

In deepfake generation studies, Lyu [2] categorised the manipulation types into three categories—head puppetry, face swapping, and lip syncing—as shown in Figure 1. Head puppetry (also called facial re-enactment [33]) involves changing the target’s entire head and upper shoulder according to the head of the source person to give the same appearance as the target. Face swapping is the process where the target’s faces are swapped with synthesised faces from the source, maintaining facial expressions. Lip syncing creates a fake video by altering the target’s lips to be consistent with speech chosen by the attacker (i.e., it “puts words into the target’s mouth”).

### 2.2. Deepfake Detection

The literature reveals a progression from early heuristic-based techniques to sophisticated architectures designed for the deepfake detection task. This section reviews the state-of-the-art studies on deepfake detection, highlighting key methodologies, datasets, and challenges in this evolving threat.

Afchar et al. [34] were the first to detect deepfake videos without using traditional image forensics techniques. They proposed the MesoNet model, a CNN architecture with a few layers focusing on the images’ mesoscopic properties (smaller semantic details) to analyse video frames. Two different types of architecture were used: Meso4 and MesoInception4. Another study carried out by Nguyen, Yamagishi, and Echizen [35] investigated the utilisation of Capsule Networks for detecting fake images and videos. Capsule Networks, recognised for their proficiency in discerning spatial hierarchies within datasets, present a promising alternative to conventional Convolutional Neural Networks (CNNs) by mitigating their shortcomings in terms of the identification of object poses and deformations. Their research leveraged the unique capabilities of Capsule Networks to improve the accuracy and robustness of detection. Dang et al. [36] proposed using an attention mechanism to produce an improved feature map, which is then used for both fake detection and predicting associated manipulation regions. Wodajo and Atnafu [37] proposed using a CNN and Vision Transformer (ViT) hybrid model to learn both local and global features. The CNN acts as a learnable feature extractor. The features are input into the ViT and classified using the attention mechanism. Zhao et al. [38] used fine-grained classification, which gathers local discriminative features to differentiate between categories in order to solve the deepfake detection problem. This model uses a multi-attentional network that includes three key components: textural feature enhancement blocks, multiple spatial attention heads, and textural and semantic features aggregation. Luo et al. [39] suggested a model for solving the generalisation problem. They found that CNN-based detectors exhibit biases to fakery method-specific textures. Since high-frequency noises remove colour textures, they proposed using these types of noise to remove the colour textures, exposing statistical discrepancies between real and fake images. Wang et al. [40] proposed a hybrid model that combines both CNN and transformer architectures. This technique is designed to overcome the shortcomings of current deepfake detection methodologies, especially regarding their generalisability across diverse datasets. The proposed model demonstrates improved performance in detecting deepfakes compared to traditional CNN-based methods—particularly in cross-dataset evaluations—achieving an AUC of 0.98 on FF++, 0.74 on DFDC, and 0.72 on Celeb-DF.

Although multiple studies have tried to address the generalisation issue for deepfake detection, there is still significant room for improvement, and the effect of using an advanced deep learning model to solve this challenge should be explored.

### 2.3. CNN-Based Approaches

CNNs are a class of deep learning models that have revolutionised fields such as computer vision and natural language processing. CNNs are designed to automatically and adaptively learn spatial hierarchies of features from input images, rendering them highly effective for tasks such as image classification, object detection, and image denoising. This capability is achieved through the use of convolutional layers, pooling layers, and fully connected layers, which together form the architecture of a CNN. The convolutional layers apply a series of filters to the input data, capturing local patterns, while pooling layers reduce dimensionality, and fully connected layers integrate the learned features for classification or regression tasks [41].

Deep learning is the dominant deepfake detection approach, with CNNs being the most represented specific architecture [42]. CNNs employ a convolution filter that extracts important edges by filtering the surrounding pixel values, independent of their position [43]. There are two types of features in images that provide different information: local and global features. Local features describe small groups of pixels (also known as image “patches”), while global features describe the entire image [44]. Even though CNNs produce outstanding performance in learning local image information, their limited receptive fields prevent them from capturing the spatial interdependence of pixels; in other words, CNN models tend to concentrate only on the activated segment of the face and ignore other parts. As a result, CNNs cannot determine and leverage the relationships between the different parts of images; for instance, the model is unable to detect an unnatural relationship between the mouth and eyes. Additionally, CNNs present an overfitting problem and cannot carry out generalisation relative to unseen fake videos during training or diverse categories of deepfake generation techniques [45].

### 2.4. ViT-Based Approaches

A transformer (a form of neural network) learns context and meaning across sequential data. It harnesses the concepts of attention or self-attention to detect the relationships between elements, even if they are far away. Before the invention of transformers, users were required to train neural networks using large, labelled datasets. It is acknowledged that the production of such datasets is resource-intensive. Transformers eliminate this need by mathematically identifying patterns between elements. Additionally, the implementation of transformer theory lends itself to the use of parallel processing, allowing these models to run quickly [46]. Moreover, transformers discover the long-term dependency between video frames and are scalable to highly complex models on large-scale datasets [47].

Transformers have achieved considerable success in natural language processing (NLP) tasks. This has inspired their application to computer vision (CV) problems, including object detection [48], image recognition [49], video classification [50], image segmentation [51], image captioning [52], and visual question answering (where the developed model must respond to questions posed about an image) [53]. They have achieved state-of-the-art results. 

**Figure 2:**
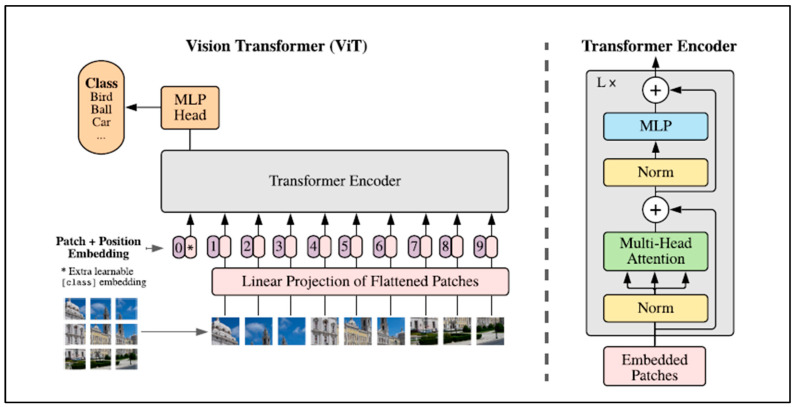
Vision Transformer (ViT) architecture. Source: [49].

The Vision Transformer (ViT) model was introduced in 2021 by Google [49]. Their model applies attention to small “patches” of the image, rather than individual pixels. As clarified in Figure 2, the ViT model divides an image into fixed-size (16 × 16 pixel) patches, flattens the patches, and includes positional embedding as an input to the transformer encoder. The encoder comprises Multi-Head Self-Attention (MSA) and Multi-Layer Perceptron (MLP) components. The model is then trained and fine-tuned for image classification. The features are linked by the Multi-Head Self-Attention Layer (MSL), which enables the information to be globally distributed across the overall image.

ViTs have two advantages over CNNs. Firstly, they have input-adaptive weighting. Unlike a convolution kernel, which is static and input-independent, their attention weights are dynamic and may change according to input. The second advantage is a global receptive field, which means that a ViT can observe the entire image in one glance (while a CNN usually does not, as mentioned above) [54].

However, transformers have some limitations. First, image attention networks often struggle with translational invariance, meaning that their performance can vary when objects in an image are shifted or repositioned. Thus, in order to outperform CNNs, ViTs had to be trained on large datasets comprising hundreds of millions of images [54]. If the ViT-based models are trained with insufficient data, they perform worse than CNNs and do not generalise well. Moreover, ViT-based models focus on global features and underperform CNNs in local features [55].

### 2.5. CoAtNet Model

**Figure 3:**
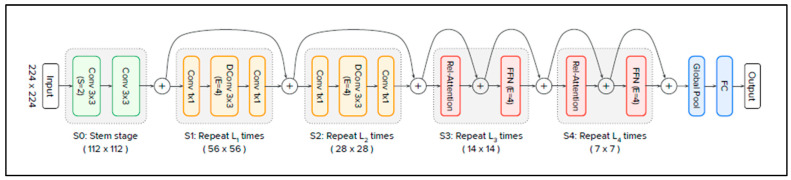
CoAtNet architecture. Source: [54].

Vision Transformers have received increasing interest in computer vision; however, they have some drawbacks. The same is true for CNNs. CoAtNet [54] seeks to combine the strengths of both. As clarified in Table 1, CoAtNet (pronounced “coat” net) is the abbreviation of convolution and self-attention, and it appeared at the end of 2021. It is a hybrid model built from ViTs and CNNs. It improves the generalisation ability, capacity, and efficiency of the model. Model generalisation refers to the ability of the model to maintain a level of performance relative to unseen data that is similar to that relative to training data. This requires the avoidance of “overfitting”. In comparison, model capacity refers to a model’s ability to accommodate large training datasets. When training data are numerous and overfitting is not a concern, the model with the higher capacity will achieve superior final performance results after an adequate training step. CoAtNets obtain state-of-the-art performance when applied to the ImageNet dataset under varying resource constraints [54]. Figure 3 presents the architecture of the CoAtNet model [54].

The architecture consists of five stages (S0, S1, S2, S3, and S4), starting with S0 and then C-C-T-T, where C represents convolution and T represents transformer. S0 is a simple two-layer convolutional stem, and it is used to lower dimensionality. S1 and S2 are convolution blocks. They contain Mobile Inverted Bottleneck Convolution (MBConv) blocks, which employ depth-wise convolution with Squeeze–Excitation (SE) to reduce the spatial size before being transferred to global attention mechanisms. S3 and S4 are transformer blocks, and they contain relative self-attention components followed by a Feed-Forward Network (FFN). Relative self-attention uses the position between patches instead of their absolute position. The latter approach is used by the standard ViT. Finally, the CoAtNet ends with global pooling and a fully connected layer.

## 3. Proposed Framework for Evaluating the Generalisation of the CoAtNet Model

The proposed framework is divided into two stages, each of which includes a set of experiments. In the first stage (Experiment Settings), the experiments explore various parameters (frame size, using a pre-trained model, data augmentation, and threshold strategies) and features (face alignment and Local Binary Pattern (LBP) features) to identify the most effective settings. These preliminary experiments serve as a foundation to determine which settings provide the best performance in the AUC of the CoAtNet model. The second stage (Performance Improvements) involves adopting these best-performing settings for the investigation of a variety of frame selection strategies: a single middle-of-video frame, fifteen random frames, fifteen optical flow frames (essentially consecutive frames allowing inter-frame relationships to be leveraged), or keyframes using cosine similarity and facial landmarks. 

**Figure 4:**
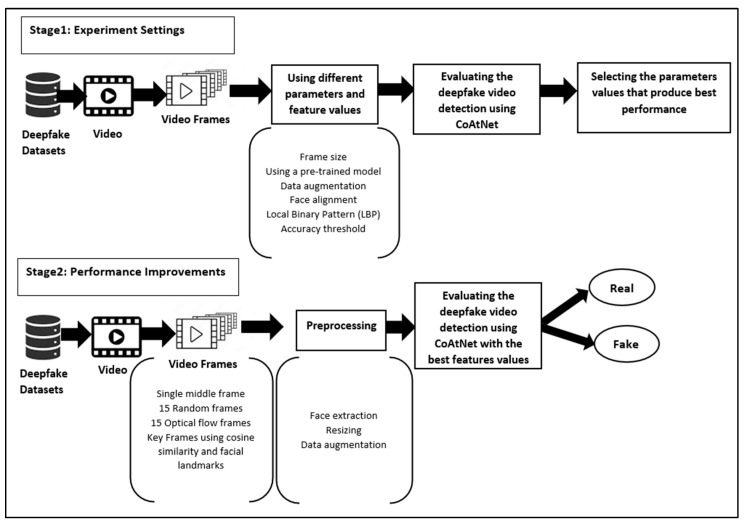
Proposed framework for evaluating the generalisation of the CoAtNet model.

Figure 4 illustrates the details of the proposed framework for evaluating the generalisation ability of the CoAtNet model. The face images are extracted from video frames using Dlib [56] to remove non-facial (background) information that is useless for deepfake detection. In fact, tracking facial information rather than using the complete frame as input should improve performance. The cropped face images are resized to 224 × 224. A particular CoAtNet implementation has been selected [57], which was designed for multiclassification over the CIFAR10 dataset [58].

### 3.1. Stage 1: Experimental Settings

This section outlines the datasets used, the proposed model, the implementation details, and the initial experiments for deciding the best combination of settings. In this stage, all models are trained on a single middle frame from each video in the DeepFakes category of FF++ instead of using the complete dataset to see the effect of different features within a reasonable time frame. 

#### 3.1.1. Datasets

The FaceForensics++ [59], DFDC [60], Celeb-DF [61], and FaceShifter [59] datasets were used in this study to ensure robust evaluations across different manipulation techniques and real-world scenarios. They provide diverse datasets with varying difficulty levels, helping to assess model generalisation for both intra-dataset and cross-dataset performance. Using these datasets also enables direct comparisons with previous studies, ensuring fair benchmarking and highlighting improvements or limitations in generalisation across datasets.

1. FaceForensics++ [27]

FaceForensics++ includes four faking algorithms: DeepFakes (DF), Face2Face (F2F), FaceSwap (FS), and NeuralTextures (NT).

- DF: This fakery approach uses two autoencoders with a shared encoder trained to reconstruct source and target face images. A face detector crops and aligns images, and the trained Encoder–Decoder of the source is applied to the target to generate a fake image. The final output is blended using Poisson image editing for seamless integration.
- F2F: This fakery approach reconstructs a 3D face model; tracks expressions, poses, and lighting; and transfers 76 Blendshape coefficients from the source to the target. The approach automates keyframe selection and re-enactment manipulation for realistic facial synthesis.
- FS: This fakery approach is a graphic-based method that transfers a face region from a source video to a target using detected facial landmarks. It fits a 3D template model with blended shapes, back-projects it onto the target, and blends the rendered model with the image, applying colour correction for a seamless result.
- NT: This is a rendering approach that learns a neural texture of the target person from video data, incorporating a rendering network trained with photometric reconstruction and adversarial losses. It uses tracked geometry during training and testing, applying patch-based GAN loss for realistic facial re-enactment.

**Figure 5:**
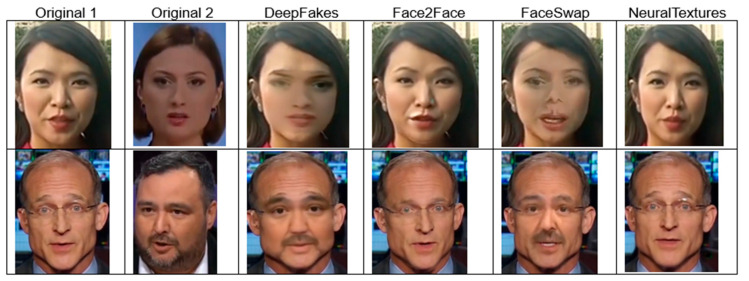
FaceForensics++ dataset examples. Source: [59].

Figure 5 shows some examples from the FF++ dataset. The first two columns (“Original 1” and “Original 2”) contain unaltered images of individuals. The following four columns display manipulated versions of the original images using the four faking techniques. We have selected two examples from the FF++ dataset presented in the first and second rows of Figure 5. Each row demonstrates how the same person appears under different manipulation methods. Each method has different visual artefacts that highlight the challenges of detecting deepfakes, as some methods seem more realistic than others.

2. DFDC [28]

The DFDC dataset contains instances created using eight faking methods: Deepfake Autoencoder (DFAE), which used two input/output resolutions of 128 × 128 (DF-128) and 256 × 256 (DF-256); Morphable Model/Neural Network (MM/NN); Neural Talking Head (NTH); FaceSwapGAN (FSGAN); StyleGAN; Refinement; and AudioSwap (Audio). The faking techniques can be integrated with one another:

- Deepfake Autoencoder (DFAE): This is a convolutional autoencoder with a shared encoder and two separately trained decoders for each identity in a face swap. It extends the shared encoder beyond the bottleneck and uses PixelShuffle for upscaling. This design helps the encoder learn common features while the decoders capture identity-specific details, enabling realistic face swaps during inference. Two resolutions are used: 128 × 128 (DF-128) and 256 × 256 (DF-256).
- Morphable Model/Neural Network (MM/NN): This method uses a frame-based morphable-mask model to perform face swaps. It aligns source and target facial landmarks, morphs source pixels to match the target, and blends the eyes and mouth from the original video. Spherical harmonics adjust illumination, and a nearest-neighbour approach selects the best source–target face pair based on expression similarity.
- Neural Talking Head (NTH): This generates realistic talking heads using few-shot and one-shot learning. The process encompasses two phases: meta-learning, which facilitates the conversion of landmarks into authentic facial representations, and fine-tuning, wherein a pre-trained model rapidly adapts to new faces. It is fine-tuned on DFDC video pairs by extracting landmarks from a driving video and generating images with the target person’s appearance.
- FaceSwapGAN (FSGAN): FSGAN uses GANs for face swapping and re-enactment, adapting to pose and expression changes. It employs adversarial loss for re-enactment and inpainting, with additional generators for face segmentation and Poisson blending.
- StyleGAN: StyleGAN performs face swaps by projecting a fixed identity descriptor onto the latent face space for each video frame, ensuring consistent identity transfer throughout the video.
- Refinement: In the final step of fake generation, a randomly selected set of videos underwent post-processing. Applying a basic sharpening filter to the blended faces significantly enhanced the visual quality of the final video, with almost no additional computational cost.
- AudioSwap (Audio): Some video clips underwent audio swapping using the TTS Skins voice conversion method [62]. TTS Skins can perform multi-voice Text-to-Speech (TTS) by converting a TTS-generated voice into various target voices.

**Figure 6:**
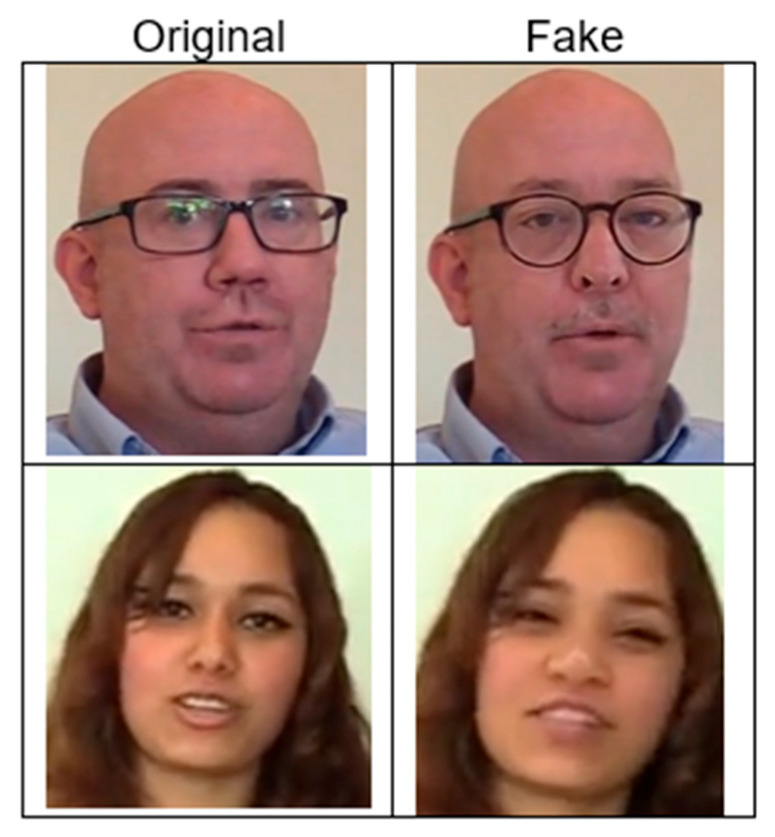
DFDC dataset examples. Source: [60].

Figure 6 presents some examples from the DFDC dataset. The left column (“Original”) contains unaltered images of individuals, while the right one (“Fake”) contains deepfake-altered versions of the same individuals. Two examples from the DFDC dataset are presented in the first and second rows. As illustrated in Figure 6, deepfake manipulations are subtle. They may be difficult to detect with the naked eye, and automated detection may need to be more sophisticated.

3. Celeb-DF [29]

**Figure 7:**
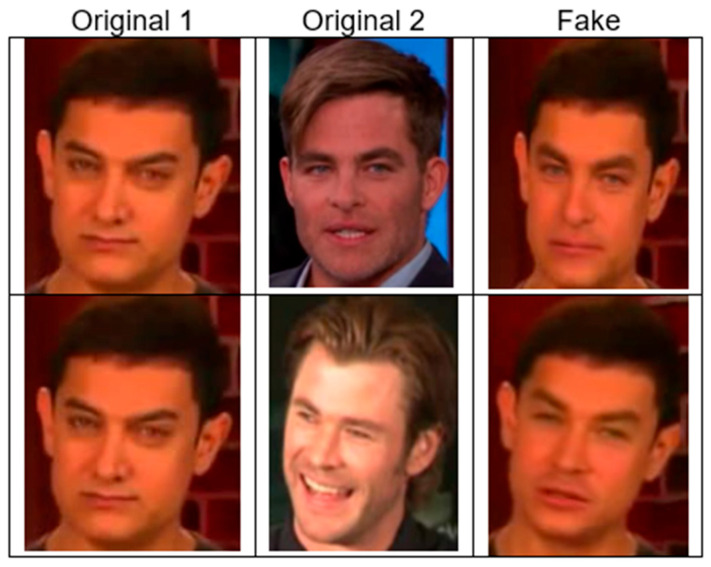
Celeb-DF dataset examples. Source: [61].

Here, the standard DeepFake generation method is refined using multiple methods to address specific visual artefacts present in existing datasets. Figure 7 presents two examples of the Celeb-DF dataset. The “Original 1” and “Original 2” columns contain unaltered images of individuals, while the next one (“Fake”) contains a fake version generated by applying face swapping between the two original individuals. Two examples are shown. It is clear that some deepfakes appear highly realistic, making detection difficult without advanced models. In the Celeb-DF dataset, there is version 1 (V1), which includes 795 videos, and version 2 (V2), which includes 5639 videos. In this study, we used Celeb-DF V2.

4. FaceShifter (FSh) [30]

**Figure 8:**
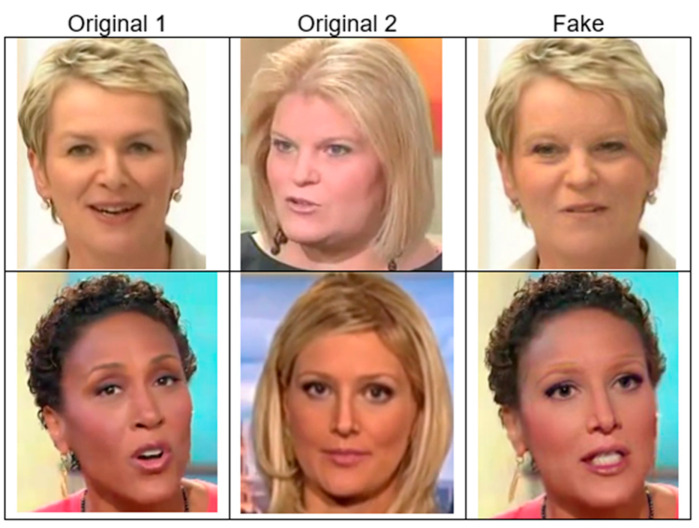
FaceShifter dataset examples. Source: [59].

FaceShifter includes a single faking method that consists of a two-stage face-swapping algorithm. Figure 8 shows two examples of the FaceShifter dataset in the first and second rows. It produces realistic deepfake data, which challenge the deepfake detection process.

Table 2 summarises the specifications of the well-known datasets. As clarified above, each dataset was created using different deepfake techniques, which implies a significant challenge in deepfake detection due to the diversity of manipulation methods and their unique artefacts. Each technique introduces distinct visual and temporal inconsistencies, rendering it difficult for a single model to carry out generalisation effectively across all types [31]. Table 3 shows the overlap between the datasets in the used faking algorithms. Although some common techniques exist, each dataset used an improved version to generate the fake data.

The datasets were split into training, validation, and testing sets with ratios of 70%, 15%, and 15% for the FF++, Celeb-DF, and FaceShifter datasets. The DFDC dataset comes pre-packaged with training, validation, and testing sets, and the research of others has respected this split. We calculated the adopted split as 93%, 3%, and 4%. The datasets are not balanced; thus, balancing was applied using oversampling for the training, validation, and testing sets.

#### 3.1.2. Proposed Model: CoAtNet16A

In this study, we introduce CoAtNet16A, a hybrid architecture that combines the convolutional–transformer design of CoAtNet with transfer learning from VGG16 weights, further enhanced by a tailored augmentation strategy. This architecture is motivated by two observations: (1) CoAtNet effectively merges the strengths of CNNs and transformers, and (2) VGG16 pretraining, while traditionally used in CNNs, has not been systematically integrated into hybrid transformer-based models such as CoAtNet for deepfake detection tasks.

To evaluate the contribution of this innovative methodology, we performed a comparative analysis involving three model configurations: (i) a CoAtNet model trained from scratch, (ii) CoAtNet pre-trained on ImageNet [63], and (iii) CoAtNet pre-trained using VGG16 weights [64], referred to as CoAtNet16. To further improve CoAtNet16, we applied several data augmentation strategies [65,66,67]. The configuration with the augmentation method from [67] is denoted as CoAtNet16A. The details of the experiment result are found in Section 3.1.3.

#### 3.1.3. Parameter Settings

In this section, we describe the parameter settings applied in our experiments, and we explain the different experiments used to explore and evaluate features and variations in deepfake video detection. The batch size that was used is 16, and the initial learning rate is 1 × 10^−3^. Moreover, the AdamW optimiser was used to train the model for 50 epochs. The code was run on an NVIDIA A100 Tensor Core GPU, which is supported by the Aziz Supercomputer operated by the Center of Excellence in High-Performance Computing [68]. The base model was trained with three frame sizes: 32 × 32, 128 × 128, and 224 × 224. According to the results in Table 4, using a frame size of 224 × 224 produced better AUC performances. In the following subsections, we present the results of three experiments for deepfake video detection. These experiments aim to determine the effectiveness of transfer learning, image transformation, and texture-based features in enhancing detection accuracy across various manipulation methods and datasets. We conducted extensive experiments; however, for the sake of clarity in the manuscript, we present the best experimental results for the following:

- Training from scratch vs. the pre-trained model.
- Image adjustment using face alignment.
- Training on Local Binary Pattern (LBP) features.

1. Results of Training from Scratch vs. Pre-trained Model

The decision to use pre-trained models in this experiment stems from the potential benefits they offer in terms of generalisation ability and performance. Training a deep learning model from scratch often requires substantial amounts of data and computational resources. However, leveraging pre-trained models is a more efficient approach, allowing knowledge learned from large-scale datasets to be transferred to a new task. This process, known as transfer learning, significantly reduces the time and resources required for training while enhancing the model’s ability to generalise across unseen data. While such pre-trained models may not have been trained for the purposes of the machine learning task at hand (in our case, fake detection), they might still be expected to usefully capture important image features (e.g., edges, textures, and some elements of facial shapes). A comparative analysis was conducted, involving three configurations: a model without pretraining, one pre-trained on CoAtNet on ImageNet [63], and a model pre-trained on VGG16 [64] (referred to as CoAtNet16).

As presented in Table 5, the pre-trained CoAtNet using VGG16 weights yielded the highest average AUC across all datasets, demonstrating its superior performance relative to the other models. In addition, several augmentation methods [65,66,67] were tested to improve the performance of the selected model. As observed in Table 5, CoAtNet16, with the augmentation method provided in [67] (CoAtNet16A), exhibits the highest performance among the others.

A classification threshold represents a specific value that indicates the manner in which a predictive model allocates class labels according to the output probabilities. In the context of binary classification, models frequently yield a probability score that reflects the likelihood of an instance being categorised as part of the positive class. The classification threshold serves as the cutoff point beyond which the instance is considered positive; conversely, instances falling below this threshold are classified as negative [69]. In this experiment, the performance of using a fixed threshold (=0.5) when calculating the accuracy for CoAtNet16A is compared with using a dynamic threshold during the training epochs. As observed in Table 6, using a static threshold produces better AUC results by about 3%. In deepfake detection, where fake and real classes often have overlapping probability distributions, dynamically adjusting the threshold results in the more frequent misclassification of borderline cases.

2. Results with and without Face Alignment

Facial alignment encompasses the identification of specified reference points on the face, including the centres of the eyes, the corners of the mouth, and the tip of the nose. A geometric transformation is calculated utilising these reference points to guarantee that the identified facial features are positioned consistently throughout all images within the dataset [70].

This experiment is applied to verify the importance of using facial alignment with CoATtNet16A. As clarified in Table 7, adding facial alignment decreases the performance slightly by about 4%. The alignment process typically includes resizing, warping, or pixel interpolation, which can smooth out key visual inconsistencies that the model could use for classification.

3. Results using Local Binary Pattern (LBP) features.

As LBP is well known for capturing texture details, it was employed to observe its effect in deepfake detection. As depicted in Table 8, using LBP almost does not affect deepfake detection when also using CoAtNet16A.

Using LBP with CoAtNet16A results in approximately the same performance as without LBP because CoAtNet already learns strong local and global features; in this context, LBP becomes redundant, and there is no need to use LBP features.

As a result of Stage 1, all features and parameters with the best performance in all cases were selected and used in the next experiments (Stage 2). The selected features and parameters are as follows: frame size: 224 × 224; using a CoAtNet model pre-trained on VGG16 with augmentation (CoAtNet16A); using a static threshold for accuracy; without using face alignment; and without using LBP features. These features and parameters are summarised in Table 9.

### 3.2. Stage 2: Performance Improvements

**Figure 9:**
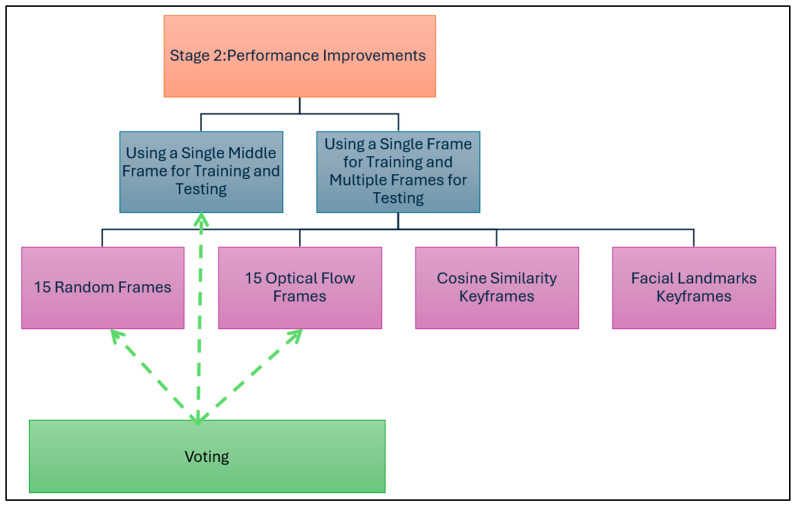
Frame selection approaches for performance improvements.

In the second stage of the experiments, the best model settings obtained from Stage 1 (Table 9) were used to apply multiple variations to improve the model’s performance. As depicted in Figure 9, the variations related to the selected frames for training and testing are either a single frame for training and testing or a single frame for training and multiple frames for testing. Finally, voting is applied to the results of the best approaches.

Frame selection is a critical step in deepfake detection. The rationale for using frame selection instead of processing all frames in a video relates to computational efficiency and avoiding redundant information. Processing every frame in a video significantly increases the computational cost and storage requirements without necessarily providing additional benefits for classification performance. This approach strikes a balance between efficiency and effectiveness, enabling the evaluation of large datasets within reasonable resource constraints [71].

Five approaches were evaluated for frame selection: a single middle frame, fifteen random frames, fifteen optical flow frames, cosine similarity keyframes, and facial landmark keyframes. For the single middle frame, the middle frame in each video was selected regardless of the video’s length. If the middle frame did not contain a face, then a search of the adjacent frames was carried out until a face was found. For fifteen random frames, fifteen random points were selected to select the frames at these points, ensuring that the selected frames cover various positions. This method aims to capture a diverse and potentially representative subset of frames. The third approach involved generating the optical flow, which is used to estimate the movement of objects between consecutive frames using Gunnar Farneback’s algorithm [72]. It works by analysing image intensity patterns at the pixel level. This method provides dense flow computations for each pixel, capturing both the direction and magnitude of motion. For this study, sixteen consecutive frames were extracted with a randomly selected initial frame, and OpenCV was utilised to calculate fifteen optical flow frames [73]. The flow is displayed in RGB channels to indicate the direction and magnitude of motion [74]. The other method for frame selection comprises the use of cosine similarity, which aims to select representative frames that summarise a video’s content, ensuring temporal and semantic diversity while reducing redundancy. This technique exploits the mathematical characteristics inherent in cosine similarity to quantify the degree of similarity among frames, thus facilitating the identification of frames that are most distinctive of the video sequence and thereby using limited representative frames instead of the entire video [75]. Finally, the last method is the extraction of facial landmark keyframes. It encompasses the identification and selection of frames from a video that exhibit prominent facial characteristics. This methodology employs facial landmark extraction techniques to ascertain critical reference points on the human face, including the eyes, nose, and mouth, which are essential for the recognition of individuals [76].

**Figure 10:**
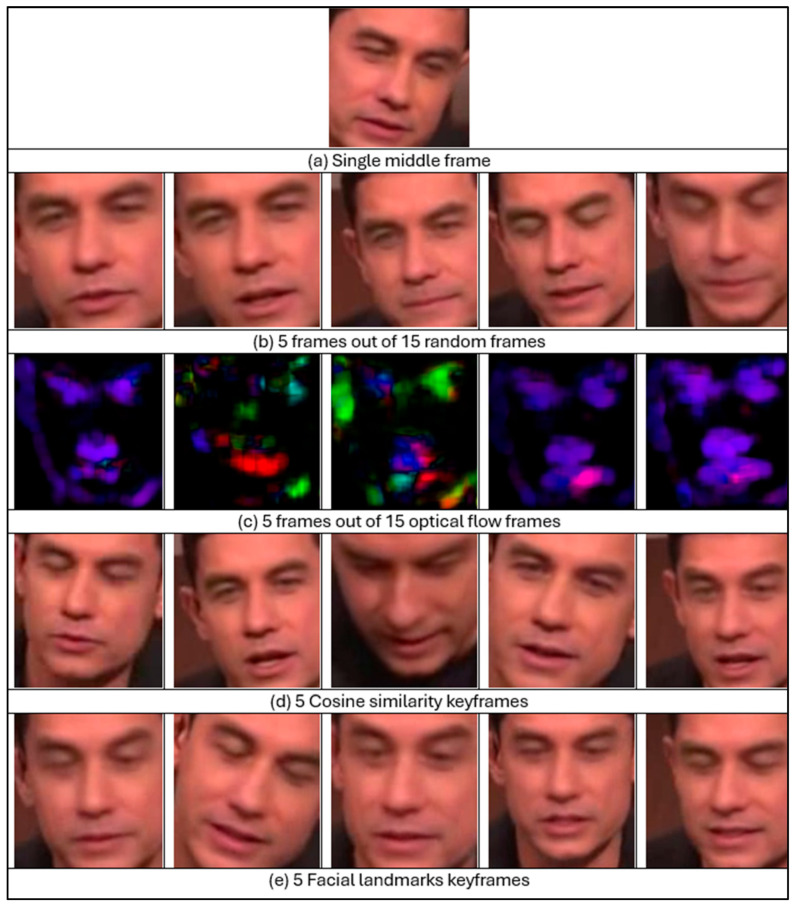
Used frame selection approaches. Frames are extracted from Celeb-DF dataset.

Figure 10 shows the different frame selection approaches used in this study.

The extracted frames using different approaches are used in training and testing via different methods, as explained in the following subsections.

#### 3.2.1. Using a Single Middle Frame for Training and Testing

In this experiment, the CoAtNet16A model was evaluated on multiple deepfake detection datasets using a single middle frame from each video. The goal was to assess the performance of CoAtNet16A when limited to only one representative frame per video, and to determine its effectiveness in detecting deepfakes under such constraints. According to Table 10, the model demonstrates varied performances across datasets, ranging from 0.63 to a maximum of 0.85 when trained on FF++.

#### 3.2.2. Using a Single Frame for Training and Multiple Frames for Testing

This part of the experiment aims to verify the effect of using multiple frames with different types. The following cases were examined: fifteen random frames, fifteen optical flow frames, cosine similarity keyframes, and finally, facial landmark keyframes. In all cases, the model was trained on single frames and tested using the voting of multiple frames.

1. Fifteen Random Frames

This experiment involved extracting fifteen random frames from each video training process as single-frame inputs and testing the voting of the fifteen random frames. As demonstrated in Table 11, training on FF++ yields the highest average AUC (0.8605). Conversely, training on datasets such as FaceShifter results in the lowest average AUC (0.5187), highlighting challenges in generalising to other datasets.

2. Fifteen Optical Flow Frames.

In this experiment, the model was trained on a single-frame optical flow extracted and then evaluated using the majority for fifteen optical flow frames. The highest average AUC is 0.8346, as illustrated in Table 12.

3. Cosine Similarity Keyframes.

This experiment investigated deepfake detection using keyframes selected based on cosine similarity. The model was trained on FF++ categories and evaluated across all datasets. The highest average AUC was about 0.85 on the FF++ dataset, as observed in Table 13.

4. Facial Landmark-Based Key Frame Selection.

Facial landmark-based key frame selection is a technique used in video analysis to identify the most informative frames in a video based on facial landmark movements [76]. This experiment explored the effect of using facial landmarks as keyframes. The model was trained on FF++ categories and evaluated across all datasets. The highest average AUC was about 0.84 on the FF++ dataset, as observed in Table 14.

Among the different types of multiple frames, the best average AUC result was obtained using fifteen random frames on the FF++ dataset, which equals approximately 0.86, as shown in Table 11.

#### 3.2.3. Using Voting for the Best Results

Among the different experiments that were applied using various settings and frame selection, the best ones were selected in order to apply the average voting technique. Our voting strategy is applied by obtaining the prediction for each item in the dataset based on the result of a specific trained model. Then, the average for different model predictions for each item is calculated. If the average is greater than or equal to 0.5, then the item is considered label 1; otherwise, it is considered label 0.

For each voting process, three models were selected, which were trained on a single middle frame, fifteen random frames, and fifteen optical flow frames; these were trained either on FF++ or only on part of FF++, called DeepFakes. As we used three trained models, there are four different combinations of voting, which comprise voting on all three models; voting on fifteen random frames and fifteen optical flow frames; voting on a single middle frame and fifteen random frames; and finally, voting on a single middle frame and fifteen optical flow frames. As depicted in Table 15, the best performance for FF++ is obtained using voting for all (single frame, fifteen random frames, and fifteen optical flow frames), which equals 0.9996; in contrast, using voting of all when trained on the DeepFakes dataset provides the best AUC for DFDC, which equals 0.5705, and for Celeb-DF, the best AUC equals 0.7624, which was obtained when using the voting of fifteen random frames and fifteen optical flow frames.

## 4. Performance Evaluation Comparison

In the experimental evaluation, various state-of-the-art methods were compared with our proposed CoAtNet models on the selected datasets—including FF++, DFDC Preview, and Celeb-DF—to assess their effectiveness in deepfake detection. The DFDC Preview dataset, a publicly available subset of the larger DFDC dataset, comprises over 5000 labelled videos, including both real and fake videos [77]. As the results of the state-of-the-art studies reported in [40] were applied to DFDC Preview and not DFDC, we adopted the same dataset to ensure comparability and the fair evaluation of our model against previously published research. The comparative performance of baseline methods and proposed CoAtNet variations is summarised in Section 4.3.

### 4.1. Intra-Dataset Comparison (Performance on FF++ Dataset)

Our proposed model, leveraging voting of all (single frame, fifteen random frames, and fifteen optical flow frames), achieved an exceptional AUC of 0.9996, outperforming all baseline methods. For example, Xception [78] and the Deep Convolutional Pooling Transformer [40] produced AUC scores of 0.9651 and 0.9766, respectively. This improvement suggests that the use of voting for all is highly effective for intra-dataset training and testing scenarios.

### 4.2. Assessment of Generalisation Through Cross-Dataset Comparison

To evaluate the generalisability of our proposed models beyond the training dataset, we conducted cross-dataset evaluations by training on FF++ and testing on two unseen datasets: DFDC Preview and Celeb-DF. This process simulates real-world scenarios where a model may encounter manipulated videos that differ in synthesis techniques from the training data. The results are organised into three subsections: the first delineating performance metrics on the DFDC dataset, the second addressing the Celeb-DF dataset, and the third one measuring the generalisation gap, followed by a comprehensive analytical summary.

#### 4.2.1. Generalisation to the DFDC Dataset

On the DFDC dataset, the performance of our model produced an AUC of 0.6781, which is the third-highest performance. In fact, this result aligns with other methods, which also exhibited performance drops when tested on DFDC. For instance, the authors of [40] achieved an AUC of 0.7368, while the authors of [78] achieved an AUC of 0.6695. These results indicate that generalisation to cross-dataset scenarios remains challenging, especially for the DFDC dataset, even for high-performing models on FF++. The reason for this is related to the different faking types, as explained in Section 3.1.1.

#### 4.2.2. Generalisation on the Celeb-DF Dataset

Our model achieved the highest performance on Celeb-DF, with an AUC of 0.7624. Simultaneously, methods such as Capsule Networks [35] and the Deep Convolutional Pooling Transformer [40] achieved AUC values of 0.6586 and 0.7243, respectively.

#### 4.2.3. Overall Generalisation

To assess the generalisation gap between the intra-dataset and cross-dataset, the following formula is used:

*Generalisation Gap* = (*performance on intra-dataset* − *performance on cross-dataset*) × 100

**Figure 11:**
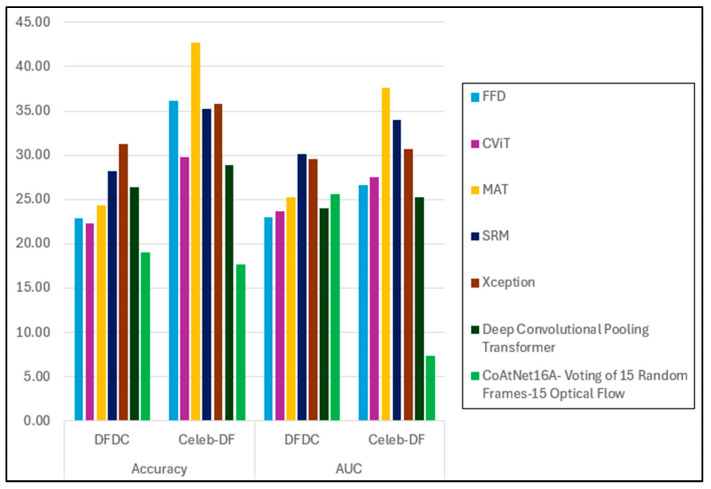
CoAtNet16A’s performance generalisation gap for cross-dataset evaluation.

For comparison with prior studies, we selected only models that achieved over 80% in both accuracy and AUC. Table 16 presents a comparison of the generalisation gap between our model (CoAtNet16A, trained on DF and using voting from 15 random RGB frames and 15 optical flow frames) and the selected methods. As shown in Figure 11, our model achieves the lowest generalisation gap in accuracy for both the DFDC and Celeb-DF datasets, with gaps of 19% and 17.7%, respectively. For the AUC, our method yields a gap of 25.6% on the DFDC dataset and achieves the best performance on Celeb-DF, with a minimal gap of 7.3%—significantly outperforming the other methods.

### 4.3. Comparative Analysis

Among all methods, the performance of our model stands out on the FF++ dataset, with an AUC of 0.9996, as in Table 17. The performance of different methods in the cross-dataset evaluations reveals, in general, a drop in both accuracy and AUC values. This observation highlights the generalisation gap in existing methods. Meanwhile, our suggested method produced the best results for the Celeb-DF dataset and the third-best result for the DFDC dataset.

**Figure 12:**
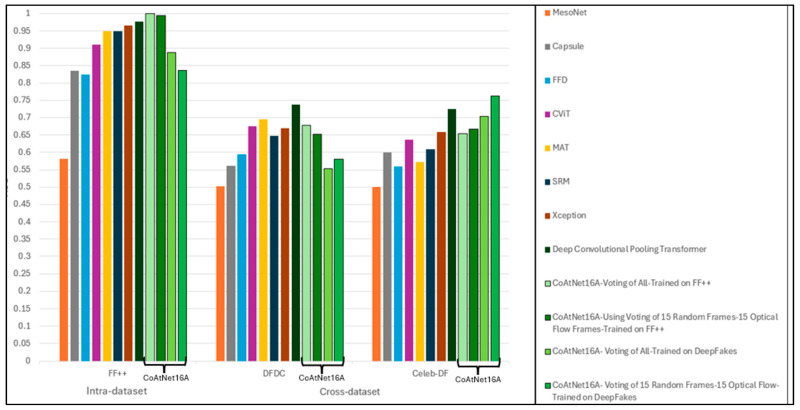
AUC scores of CoAtNet16A model variants compared to state-of-the-art deepfake detectors.

The performance comparison between the proposed CoAtNet16A model and existing baseline methods across multiple datasets is illustrated in Figure 12.

## 5. Discussion and Limitations

The performance evaluation results presented in this study demonstrate that our proposed model, CoAtNet16A—particularly the “Voting of All” variant—achieves superior accuracy and AUC scores on the FF++ dataset, outperforming state-of-the-art methods such as Xception [78] and the Deep Convolutional Pooling Transformer [40]. This performance underscores the strength of multi-frame and multi-modal voting strategies for intra-dataset detection tasks.

In cross-dataset evaluations, the performance of our models exhibited a noticeable decline, particularly relative to the DFDC dataset. Despite being among the top-performing models, our best AUC was 0.6781. This reflects a trend in deepfake detection research, where generalisation across datasets remains a significant challenge due to variations in deepfake generation techniques. Conversely, the model performed relatively well on the Celeb-DF dataset, achieving an AUC of 0.7624 and outperforming notable baselines such as Xception [78] and the Deep Convolutional Pooling Transformer [40].

These findings highlight two key contributions of this study: first, an ensemble voting strategy with CoAtNet16A that enhances performance in intra-dataset contexts and, second, the best performance on Celeb-DF, suggesting that our model captures subtle, generalisable deepfake characteristics better than some existing methods.

However, this study also has several limitations:

- Cross-dataset generalisation: Despite improvements, our model, similarly to others, exhibits performance degradation when applied to datasets on which it was not trained. This underscores the need for models capable of learning more generalised deepfake features that are invariant across datasets.
- Computational complexity: The ensemble approach, while effective, increases computational requirements due to the processing of multiple frames and modalities. This may hinder its applicability for real-time detection or on resource-constrained devices.

## 6. Conclusions

This study assessed the generalisation ability of the CoAtNet model in deepfake video detection using both intra-dataset and cross-dataset evaluations. Our strongest finding is that CoAtNet16A achieved an AUC of 0.9996 on the FaceForensics++ dataset, outperforming existing state-of-the-art models such as Xception (0.9651) and the Deep Convolutional Pooling Transformer (0.9766). For the cross-dataset scenario, our model attained the highest AUC of 0.7624 on the Celeb-DF dataset and a third-highest AUC of 0.6781 on the DFDC Preview dataset, demonstrating superior generalisation across different manipulation techniques and source distributions.

The media and content verification sector, especially in the context of journalism, law enforcement, and social media platforms, is poised to gain the most significant advantages from implementing advanced deepfake detection solutions. Automated and precise detection instruments, such as CoAtNet16A, can contribute to the preservation of public trust by identifying synthetic or manipulated content before it goes viral.

The most vulnerable sectors encompass politics, finance, and public safety, where deepfakes can be exploited for misinformation, impersonation, and fraudulent activities. Esteemed individuals, organisations, and platforms may encounter both reputational and legal risks. To mitigate the potential for such exploitation, it is imperative to implement a comprehensive defence strategy that incorporates advanced deepfake detection systems, user awareness initiatives, and transparent content origin verification.

Future research should explore the common features of various faking techniques that can be used to enhance the detection model. These developments are essential for creating trustworthy deepfake detection algorithms that can handle the quickly changing synthetic media ecosystem and guarantee their effectiveness in real-world applications.

## Figures and Tables

**Table 1:** Comparison between CNN, ViT, and CoAtNet based on source [54].

Properties	CNN	ViT	CoAtNet
Translation Equivariance	✔		✔
Local Features	✔		✔
Input-adaptive Weighting		✔	✔
Global Features		✔	✔

**Table 2:** Well-known public datasets.

Dataset	Published Date	Images/Videos	Real Videos	Fake Videos	Number of Faking Methods	Faking Algorithm Type(s)	Visual/Audio/Both
FF++	Jan-19	Videos	1000	4000	4	DF, F2F, FS, NT	Visual
Celeb-DF (v2)	Nov-19	Videos	590	5639	1	Improved DeepFake synthesis algorithm	Visual
FSh	Jun-20	Videos	1000	1000	1	Two-stage FaceShifter face-swapping	Visual
DFDC	Jun-20	Videos	23,954	104,500	8	DF-128, DF-256, MM/NN, NTH, FSGAN, StyleGAN, Refinement, and AudioSwaps	Both

**Table 3:** Overlapping of deepfake datasets.

Faking Type	FF++	Celeb-DF	FSh	DFDC
Face Swap	Yes (DeepFakes, FaceSwap)	Yes (improved version)	Yes (improved version)	Yes (DFAE, MM/NN, StyleGAN)
Head Puppetry	Yes (Face2Face, NeuralTextures)	No	No	Yes (FSGAN, NTH)
Lip Syncing	No	No	No	Yes (AudioSwap)

**Table 4:** Model performance (AUC) with different frame sizes. The models were trained on DeepFakes.

Frame Size	DF	F2F	FS	NT	DFDC	Celeb-DF	FSh	AVG AUC
32 × 32	0.9281	0.6163	0.7138	0.6245	0.6399	0.632	0.6987	0.6933
128 × 128	0.9836	0.8639	0.5168	0.8787	0.5931	0.6243	0.7262	0.7409
224 × 224	0.9977	0.9846	0.5952	0.9827	0.5423	0.4901	0.9824	0.7964

**Table 5:** Comparison of average AUC scores across different pre-trained models. The models were trained on DeepFakes.

Type	DF	F2F	FS	NT	DFDC	Celeb-DF	FSh	AVG AUC
No Pretraining	0.9940	0.9482	0.4490	0.8886	0.5137	0.4563	0.7468	0.7138
Pre-trained CoAtNet	0.9944	0.9515	0.4847	0.9170	0.4858	0.4596	0.7658	0.7227
CoAtNet16	0.9974	0.9470	0.4917	0.9068	0.5010	0.4886	0.7325	0.7236
CoAtNet16A	0.9977	0.9846	0.5952	0.9827	0.5423	0.4901	0.9824	0.7964

**Table 6:** Model performance (AUC) with static threshold (STh) and dynamic threshold (DTh). The model is trained on DeepFakes.

Threshold Type	DF	F2F	FS	NT	DFDC	Celeb-DF	FSh	AVG AUC
CoAtNet16A with STh	0.9977	0.9846	0.5952	0.9827	0.5423	0.4901	0.9824	0.7964
CoAtNet16A with DTh	0.9973	0.9686	0.5444	0.9669	0.5165	0.4867	0.8997	0.7686

**Table 7:** Model performance (AUC) with and without facial alignment. The model is trained on DeepFakes.

Face Alignment Status	DF	F2F	FS	NT	DFDC	Celeb-DF	FSh	AVG AUC
CoAtNet16A without Face Alignment	0.9977	0.9846	0.5952	0.9827	0.5423	0.4901	0.9824	0.7964
CoAtNet16A with Face Alignment	0.9986	0.9270	0.4793	0.9342	0.5691	0.5921	0.8093	0.7585

**Table 8:** Model performance (AUC) with and without Local Binary Pattern (LBP) features. The models were trained on DeepFakes.

Using LBP Status	DF	F2F	FS	NT	DFDC	Celeb-DF	FSh	AVG AUC
CoAtNet16A without LBP	0.9977	0.9846	0.5952	0.9827	0.5423	0.4901	0.9824	0.7964
CoAtNet16A with LBP	0.9997	0.9805	0.7316	0.9819	0.5003	0.5620	0.8518	0.8011

**Table 9:** Selected features and parameters with CoAtNet16A.

Frame Size	Transfer Learning	Thresholding	Image Transformation	Texture-Based Features
224 × 224	CoAtNet16A (CoAtNet model pre-trained on VGG16 with augmentation)	Static	No	No

**Table 10:** Performance (AUC) of the CoAtNet16A model on various deepfake detection datasets using a single middle frame per video.

Trained on/Tested on	DF	F2F	FS	NT	DFDC	Celeb-DF	FSh	AVG AUC FF++	AVG AUC All DS
DF	0.9977	0.9846	0.5952	0.9827	0.5423	0.4901	0.9824	0.8901	0.7964
F2F	0.9653	0.9966	0.877	0.9489	0.4975	0.5401	0.9695	0.9470	0.8278
FS	0.5558	0.8503	0.9812	0.461	0.5046	0.5339	0.5425	0.7121	0.6328
NT	0.995	0.992	0.6493	0.9952	0.4912	0.5001	0.948	0.9079	0.7958
DFDC	0.9497	0.7101	0.79	0.7059	0.8135	0.7473	0.6953	0.7889	0.7731
Celeb-DF	0.8244	0.6577	0.4463	0.6683	0.6107	0.9439	0.5395	0.6492	0.6701
FSh	0.8227	0.7855	0.5564	0.7984	0.5187	0.5243	0.9963	0.7408	0.7146
FF++	0.998	0.9976	0.9971	0.9973	0.4757	0.4924	0.9948	0.9975	0.8504

**Table 11:** Model performance (AUC) with training on a single frame and testing for fifteen random frames.

Trained on/Tested on	DF	F2F	FS	NT	DFDC	Celeb-DF	FSh	AVG AUC FF++	AVG AUC All DS
DF	0.9998	0.8401	0.3345	0.9261	0.562	0.6735	0.7744	0.7751	0.7301
F2F	0.7055	0.9864	0.5841	0.6165	0.5029	0.5639	0.5573	0.7231	0.6452
FS	0.395423	0.798824	0.996293	0.320276	0.503374	0.561768	0.409333	0.6277	0.5766
NT	0.9685	0.8567	0.4213	0.9637	0.4723	0.4839	0.7488	0.8026	0.7022
DFDC	0.9401	0.6765	0.8128	0.6732	0.8492	0.8455	0.6207	0.7757	0.774
Celeb-DF	0.8126	0.6224	0.4396	0.6321	0.6362	0.9733	0.5571	0.6267	0.6676
FSh	0.4729	0.4476	0.303	0.4257	0.5109	0.476	0.9948	0.4123	0.5187
FF++	0.9988	0.996	0.9955	0.9957	0.5547	0.5167	0.9664	0.9965	0.8605

**Table 12:** Model performance (AUC) with training on a single optical flow frame and testing for fifteen optical flow frames.

Trained on/Tested on	DF	F2F	FS	NT	DFDC	Celeb-DF	FSh	AVG AUC FF++	AVG AUC All DS
DF	0.9978	0.7838	0.5803	0.8272	0.5407	0.7352	0.6975	0.7973	0.7375
F2F	0.8013	0.9422	0.4324	0.7725	0.4647	0.5601	0.4161	0.7371	0.6270
FS	0.8513	0.6492	0.9912	0.6743	0.5460	0.6564	0.5339	0.7915	0.7003
NT	0.9015	0.8372	0.5272	0.9363	0.5014	0.6183	0.7031	0.8006	0.7179
FF++	0.9846	0.9537	0.9787	0.9292	0.5445	0.7488	0.7029	0.9615	0.8346

**Table 13:** Model performance (AUC) with cosine similarity keyframes.

Trained on/Tested on	DF	F2F	FS	NT	DFDC	Celeb-DF	FSh	AVG AUC FF++	AVG AUC All DS
DF	0.9990	0.9239	0.3514	0.9358	0.5279	0.5850	0.6865	0.8025	0.7157
F2F	0.9275	0.9997	0.4729	0.7106	0.4705	0.5541	0.5501	0.7777	0.6693
FS	0.5743	0.6947	0.9993	0.5862	0.4810	0.5206	0.5883	0.7136	0.6349
NT	0.9899	0.9348	0.4623	0.9812	0.5166	0.5338	0.8150	0.8420	0.7477
FF++	0.9968	0.9930	0.9889	0.9853	0.5592	0.5486	0.8769	0.9910	0.8498

**Table 14:** Model performance (AUC) with facial landmark keyframes.

Trained on/Tested on	DF	F2F	FS	NT	DFDC	Celeb-DF	FSh	AVG AUC FF++	AVG AUC All DS
DF	0.9991	0.8903	0.3779	0.9382	0.5137	0.4941	0.8462	0.8014	0.7228
F2F	0.9601	0.9983	0.5467	0.8545	0.5217	0.5140	0.6242	0.8399	0.7171
FS	0.6011	0.5760	0.9979	0.5714	0.5149	0.4813	0.5287	0.6866	0.6102
NT	0.9976	0.9627	0.4518	0.9944	0.5096	0.4058	0.9525	0.8516	0.7535
FF++	0.9994	0.9987	0.9988	0.9960	0.5083	0.4277	0.9331	0.9982	0.8374

**Table 15:** Voting performance on the CoAtNet16A model (ACC: accuracy; AUC: area under the curve).

Frames Type	Trained On	Testing Datasets					
		FF++		DFDC		Celeb-DF	
		ACC	AUC	ACC	AUC	ACC	AUC
Single Frame	FF++	98.1618	0.9975	49.5386	0.4757	49.4681	0.4924
15 Random Frames		97.5262	0.9965	53.3404	0.5547	70.5822	0.5167
15 Optical Flow Frames		90.4332	0.9615	51.6337	0.5445	33.0604	0.7488
Voting of All		99.0584	0.9996	50.9055	0.5373	52.9621	0.6531
Voting of 15 Random Frames and 15 Optical Flow		98.1227	0.9943	52.1201	0.5655	61.8483	0.6664
Voting of Single Frame and 15 Random Frames		98.5581	0.9996 *	50.5080	0.5229	48.5782	0.5032
Voting of Single Frame and 15 Optical Flow Frames		98.6209	0.9990	50.3754	0.5123	51.0071	0.7233
Single Frame	DF	83.5784	0.8901	52.6426	0.5423	49.5272	0.4901
15 Random Frames		75.4671	0.7751	50.3095	0.5620	17.8754	0.6735
15 Optical Flow Frames		69.5274	0.7973	49.9667	0.5407	15.6602	0.7352
Voting of All		71.8410	0.8867	50.4196	0.5705 *	50.5332	0.7033
Voting of 15 Random Frames and 15 Optical Flow		68.4662	0.8353	50.2650	0.5529	50.7701	0.7624 *
Voting of Single Frame and 15 Random Frames		78.1067	0.8658	50.9717	0.5674	51.1848	0.6005
Voting of Single Frame and 15 Optical Flow Frames		79.3635	0.8965	50.5300	0.5643	50.2962	0.6782

* Bold indicates highest AUC result.

**Table 16:** Comparison of generalisation gap between CoAtNet16A and other studies.

Method	Accuracy		AUC	
	DFDC	Celeb-DF	DFDC	Celeb-DF
FFD [36]	22.85	36.10	23.01	26.62
CViT [37]	22.29	29.79	23.65	27.48
MAT [38]	24.34	42.72	25.29	37.65
SRM [39]	28.24	35.22	30.13	34.03
Xception [78]	31.31	35.84	29.56	30.65
Deep Convolutional Pooling Transformer [40]	26.35	28.84	23.98	25.23
CoAtNet16A-Voting of 15 Random Frames-15 Optical Flow	19.00	17.70	25.56	7.29

**Table 17:** ACC and AUC performance comparisons on each testing set after training on the FF++ dataset and the results of previous studies reported in [40].

Method	Trained on						
		FF++		DFDC Preview		Celeb-DF	
		ACC	AUC	ACC	AUC	ACC	AUC
MesoNet [34]	FF++	61.03	0.5813	50.02	0.5016	36.73	0.5001
Capsule [35]	FF++	76.4	0.8344	51.3	0.5616	61.96	0.5993
FFD [36]	FF++	82.29	0.8248	59.44	0.5947	46.19	0.5586
CViT [37]	FF++	83.05	0.9108	60.76	0.6743	53.26	0.636
MAT [38]	FF++	87.5	0.9485	63.16	0.6956	44.78	0.572
SRM [39]	FF++	88.17	0.9493	59.93	0.648	52.95	0.609
Xception [78]	FF++	90.08	0.9651	58.77	0.6695	54.24	0.6586
Deep Convolutional Pooling Transformer [40]	FF++	92.11	0.9766	65.76	0.7368 *	63.27	0.7243
CoAtNet16A—Voting of All	FF++	99.0584	0.9996 *	51.6129	0.6781	52.9621	0.6531
CoAtNet16A—Voting of 15 Random Frames and 15 Optical Flow Frames	FF++	98.1227	0.9943	53.4946	0.6515	61.8483	0.6664
CoAtNet16A—Voting of All	DF	71.8410	0.8867	49.7312	0.5530	50.5332	0.7033
CoAtNet16A—Voting of 15 Random Frames and 15 Optical Flow	DF	68.4662	0.8353	49.4624	0.5797	50.7701	0.7624 *

* Bold indicates highest AUC result.

## Data Availability

Forensics++ and FaceShifter datasets are available under a non-commercial research licensing agreement [79]. Celeb-DF can also be accessed by agreement [80]. DFDC was released by Facebook and can be accessed freely [60] after creating an AWS account [81] and an IAM user account [82].

## Abbreviations

The following abbreviations are used in this manuscript:

GAN	Generative Adversarial Network
CV	Computer Vision
DNN	Deep Neural Network
AE	Autoencoder
CNN	Convolutional Neural Network
ViT	Vision Transformer
ED	Encoder–Decoder
NLP	Natural Language Processing
MSA	Multi-Headed Self-Attention
MLP	Multi-Layer Perceptron
FFN	Feed-Forward Network
LBP	Local Binary Pattern
FF++	FaceForensics++
DF	DeepFakes
F2F	Face2Face
FS	FaceSwap
NT	NeuralTextures
DFDC	DeepFake Detection Challenge
DFAE	Deepfake Autoencoder
MM/NN	Morphable Model/Neural Network
NTH	Neural Talking Head
FSGAN	FaceSwapGAN
TTS	Text-to-Speech
FSh	FaceShifter
AUC	Area Under the Curve
